# Phylogenetic position of the enigmatic starfish family Podosphaerasteridae (Asteroidea, Valvatida) with a morphological observation of the skeletal structure by micro-CT

**DOI:** 10.3897/BDJ.14.e194006

**Published:** 2026-08-31

**Authors:** Shimpei F. Hiruta, Iraru Kobayashi, Toshihiko Fujita

**Affiliations:** 1 The Mt. Fuji Institute for Nature and Biology, Showa Medical University, Fujiyoshida, Japan The Mt. Fuji Institute for Nature and Biology, Showa Medical University Fujiyoshida Japan; 2 National Museum of Nature and Science, Tsukuba, Japan National Museum of Nature and Science Tsukuba Japan; 3 Research Center for Inland Seas, Kobe University, Awaji, Japan Research Center for Inland Seas, Kobe University Awaji Japan; 4 The University of Tokyo, Tokyo, Japan The University of Tokyo Tokyo Japan

**Keywords:** Asteroidea, micro-CT, mitochondrial gene rearrangement, molecular phylogeny, *
Podosphaeraster
*, Valvatida

## Abstract

Background

The genus *Podosphaeraster* comprises seven species, characterised by a distinctive spherical body, all currently known from the seabed at depths below approximately 70 m. Its peculiar morphology has made its phylogenetic placement a subject of ongoing debate. It was initially suggested to be placed in Sphaerasteridae, the same family as fossil species. However, subsequent detailed skeletal analyses of the fossil forms suggested that this similarity was likely due to convergent evolution. Recent molecular analyses have revealed that Valvatida, in which the genus is currently placed, is likely a large polyphyletic group, leaving its taxonomic position still unresolved.

New information

A detailed examination of the internal skeletal structure of *Podosphaeraster toyoshiomaruae*, collected from the seas around Japan, was conducted using micro-focus X-ray computed tomography. Concurrently, shotgun sequencing was performed to identify key molecular markers for recent asteroid phylogeny. Additionally, shotgun sequencing determined the complete mitochondrial genome. Despite conservative evolution amongst asteroidean mitochondrial genomes, a translocation of the *COX2* gene was revealed, representing the first discovery of a major protein-coding gene translocation within Asteroidea.

In the phylogenetic tree, *P. toyoshiomaruae* was positioned as the most basal lineage within Valvatida. However, the statistical support for this placement was low, potentially due to the long-branch attraction caused by the excessively rapid evolutionary rate.

Images reconstructed by micro-CT confirmed the presence of calcified reinforcement in the mesentery and showed its detailed structure for the first time.

The mesentery skeleton was found to connect to the V-plate and five pairs of plates, including three kinds of marginal plates. This suggests that the marginal plates of this species may not be homologous with those of other asteroids.

Although varying degrees of marginal plate reduction are shared with the order Velatida, we consider this to be a case of convergent evolution. Our phylogenetic analysis indicates a close relationship between *P. toyoshiomaruae* and Poraniidae (and other Valvatida), all of which possess marginal plates differentiated to varying extents, the homology of which remains uncertain.

## Introduction

The genus *Podosphaeraster* is characterised by a distinctive spherical body, with seven species currently known from the seabed at depths below approximately 70 m (Fujita and Rowe 2002, Mah et al. 2026). The phylogenetic placement of this genus, with its unusual morphology, has been a subject of ongoing debate. An initial suggestion placed it within Sphaerasteridae, originally established for fossil species (Clark and Wright 1962). Subsequent detailed skeletal analyses of the fossil forms indicated that this similarity was likely a result of convergent evolution, leading to the establishment of a new family, Podosphaerasteridae, within the order Valvatida (Fujita and Rowe 2002). However, the controversy has persisted in recent years and Gale (2021) has since re-instated the genus *Podosphaeraster* to Family Sphaerasteridae. With the description of a new species from Western Australia, the genus was classified within the family Podosphaerasteridae (Mah et al. 2026). In addition, phylogenetic analyses incorporating molecular data have revealed that Valvatida, in which this genus is currently placed, is likely a large polyphyletic group (Mah and Foltz 2011), leaving its taxonomic position still unresolved. Moreover, despite their peculiar external morphology, detailed information on the internal structure of *Podosphaeraster* species has been lacking to date.

To address this knowledge gap, we conducted a detailed micro-CT examination of the internal skeletal structure of *Podosphaeraster toyoshiomaruae*, collected from the seas around Japan. Concurrently, we performed shotgun sequencing using next-generation sequencing (NGS) to determine molecular markers used in previous phylogenetic studies and the complete mitochondrial genome sequence.

Remarkably, our analyses revealed that *P. toyoshiomaruae* possesses a highly distinctive mitochondrial genome structure, unlike the other asteroid taxa. In this paper, we present a comprehensive description of the internal morphology and genomic architecture of *P. toyoshiomaruae* and we offer new insights into the enigmatic phylogenetic position of the genus *Podosphaeraster*, based on these findings.

## Material and methods

### Molecular analyses

One specimen of *Podosphaeraster toyoshiomaruae* was used for molecular analyses. It was collected from the Oshima-shinsone Bank (29°46.57'N, 130°23.47'E), south-western Japan, at the depths of 147–149 m, on 23 May 2019. It is deposited at the National Museum of Nature and Science (NSMT E-14365). Total genomic DNA was extracted from a part of the tissue between ambulacral grooves using a DNeasy Blood & Tissue Kit (Qiagen). The sample was homogenised in 360 μl ATL buffer, then 40 μl of protease K solution was added. Overnight incubation was conducted to lyse the tissue. In the elution step, buffer EB (Qiagen) was used instead of buffer AE.

The total DNA was quantified by Qubit 4 (Thermo Fisher Scientific) with a dsDNA HS Assay Kit. A Collibri ES DNA library Prep Kit for Illumina system with UD indices (Thermo Fisher Scientific) was used to make the shotgun library. The amount of input DNA was 30 ng and the library target was 300–800 bp. The fragmentation step was set to 37°C for 10 min. The library amplification was conducted for 12 cycles. The other procedures were performed according to the manufacturer's method. The shotgun library was quantified by Qubit 4 and qualified by TapeStation 4200 (Agilent) with a D1000 DNA Assay Kit. Paired-end sequencing (300 cycles) was conducted by the HiSeq X system (Illumina).

The resulting fastq files were filtered using fastp v. 0.23.2 (Chen et al. 2018) and subsequent processing was performed in CLC Genomics Workbench 12 (Qiagen). The de novo assembly was conducted with default settings and contigs including target regions were selected using nucleotide BLAST search (McGinnis and Madden 2004, Camacho et al. 2009), both implemented in CLC Genomics Workbench. To retrieve the *histone H3* sequence of *P. toyoshiomaruae*, the map-read function was performed using the sequence of *Porania pulvillus* (EU707658) as the reference.

Annotation of the mitochondrial genome was initially performed using MITOS2 (Bernt et al. 2013, Donath et al. 2019) and some protein-coding genes (PCGs) were subsequently confirmed manually. Nuclear and mitochondrial rRNA genes were manually identified by their boundaries using the RNAfold WebServer (Lorenz et al. 2011). The codon usage in 13 PCGs was calculated by MEGA7 (Kumar et al. 2016).

To elucidate the phylogenetic position of the *Podosphaeraster* amongst Asteroid starfish, mitochondrial 12S rRNA (*rrnS*) and 16S rRNA (*rrnL*) and nuclear *histone H3* gene sequences of 117 OTUs were obtained from GenBank (Table 1). A total of 345 sequences from the three gene regions were aligned using ClustalW (Thompson et al. 1994) in MEGA7. In this dataset, sequences belonging to Velatida were treated as the out-group. For *rrnS* and *rrnL* gene sequences, a multiple alignment was performed to account for their secondary structure using MAFFT v. 7.505 (Katoh and Standley 2013) with the X-INS-i option. We performed the Substitution Saturation Test in DAMBE v. 7.3.6 (Xia 2018) to assess whether the sequences in the three-gene dataset were saturated.

PartitionFinder v. 2.1.1 (Lanfear et al. 2016) was used to determine the best partitioning scheme and substitution model for RAxML-NG v. 1.2.2 (Kozlov et al. 2019) and MrBayes v. 3.2.7 (Huelsenbeck and Ronquist 2001, Ronquist and Huelsenbeck 2003) using linked branch lengths and a greedy search algorithm (Lanfear et al. 2012). The optimal partitioning scheme and evolutionary models used in both analyses are shown in Table 2. Bootstrap analyses (Felsenstein 1985) were performed on 1,000 pseudoreplicates for the ML tree. For Bayesian analyses, the Markov-chain Monte Carlo process used random starting trees and involved two runs of four chains each for 20 million generations. Trees were sampled every 100^th^ generation; the first 25% of trees were discarded as burn-in. Convergence was inferred when the standard deviation of split frequencies became < 0.01 and the potential scale reduction factors were around 1.0. In addition, Tracer v. 1.7 (Rambaut et al. 2018) was used to ensure that parameter values had an effective sample size > 200.

### Morphological observations

Another specimen of *Podosphaeraster toyoshiomaruae* was used for the observation of the skeleton by micro-CT. It was collected between Okinawa-jima Island and Miyako-jima Island, Japan (25°33.21'N, 126°12.77'E), south-western Japan, at depths of 95–100 m, on 24 April 2002 and is deposited at the National Museum of Nature and Science (NSMT E-13935A). The specimen was scanned with a micro-CT scanner (inspeXio SMX-225CT FPD HR, Shimadzu) at NSMT. The scanning was performed at a tube voltage of 115 kV and a tube current of 70 µA for 40 minutes. Two- and three-dimensional images were reconstructed using VGSTUDIO Max v. 3.2 (Volume Graphics).

The terminology of skeletal plates in this study primarily follows Fujita and Rowe (2002) for ease of plate identification, although Gale (2021) has abandoned the terms “apical system” and “marginal plate” for this genus. We also follow the terminology for unpaired actinal plates, V- and I-plate, used by Gale (2021).

## Data resources

The data that support the findings of this study are openly available in the DDBJ/EMBL/GenBank databases under accession numbers LC913548–LC913551 for mitochondrial genome and nucleic DNA sequences. Additionally, the volumetric image data reconstructed from micro-CT is available at MorphSource (https://www.morphosource.org/) under Media ID 000810720.

## Results

### Mitochondrial genome structure

**Figure 1.**
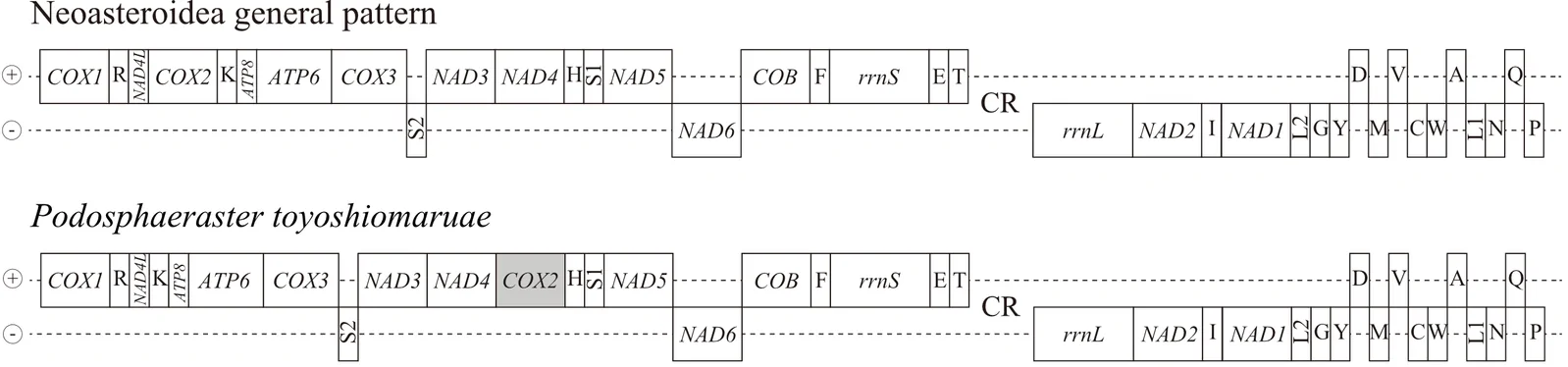
Mitochondrial gene arrangement of *Podosphaeraster toyoshiomaruae* and the general pattern for Neoasteroidea. The *COX2* gene, highlighted in grey, has translocated from the general pattern. See Table 3 for the details of the genome structure of *P. toyoshiomaruae*. The 22 transfer RNA genes are represented by the corresponding amino acid's letter. CR: control region.

The complete mitochondrial genome of *Podosphaeraster toyoshiomaruae* was determined to be 16,247 bp in length. Its nucleotide composition was AT-rich (A+T = 65.5%; G+C = 34.5%). Gene arrangement and primary structure of the mitogenome are summarised in Table 3. The start and stop codons for the 13 protein-coding genes (PCGs) followed patterns common amongst Asteroidea (Quek et al. 2020). Three genes (*NAD3*, *NAD4L* and *COB*) utilised ATT as the start codon, while the remaining ten used ATG. For the stop codons, four genes terminated with TAG, while the others ended with TAA. The codon usage frequency and relative synonymous codon usage (RSCU) values for the 13 PCGs are summarised in Table 4. Compared to the general gene arrangement known for Neoasteroidea (e.g. Payne et al. (2023)), the *COX2* gene was translocated in the *Podosphaeraster toyoshiomaruae* mitogenome and the arrangement of all other genes, including tRNAs, was identical to the general pattern (Fig. 1). A motif sequence corresponding to the origin of replication was identified in not only the control region (CR), but also the short intergenic region (118 bp), located between the *rrnL* and *NAD2* genes.

### Phylogenetic position of Podosphaeraster toyoshiomaruae

**Figure 2.**
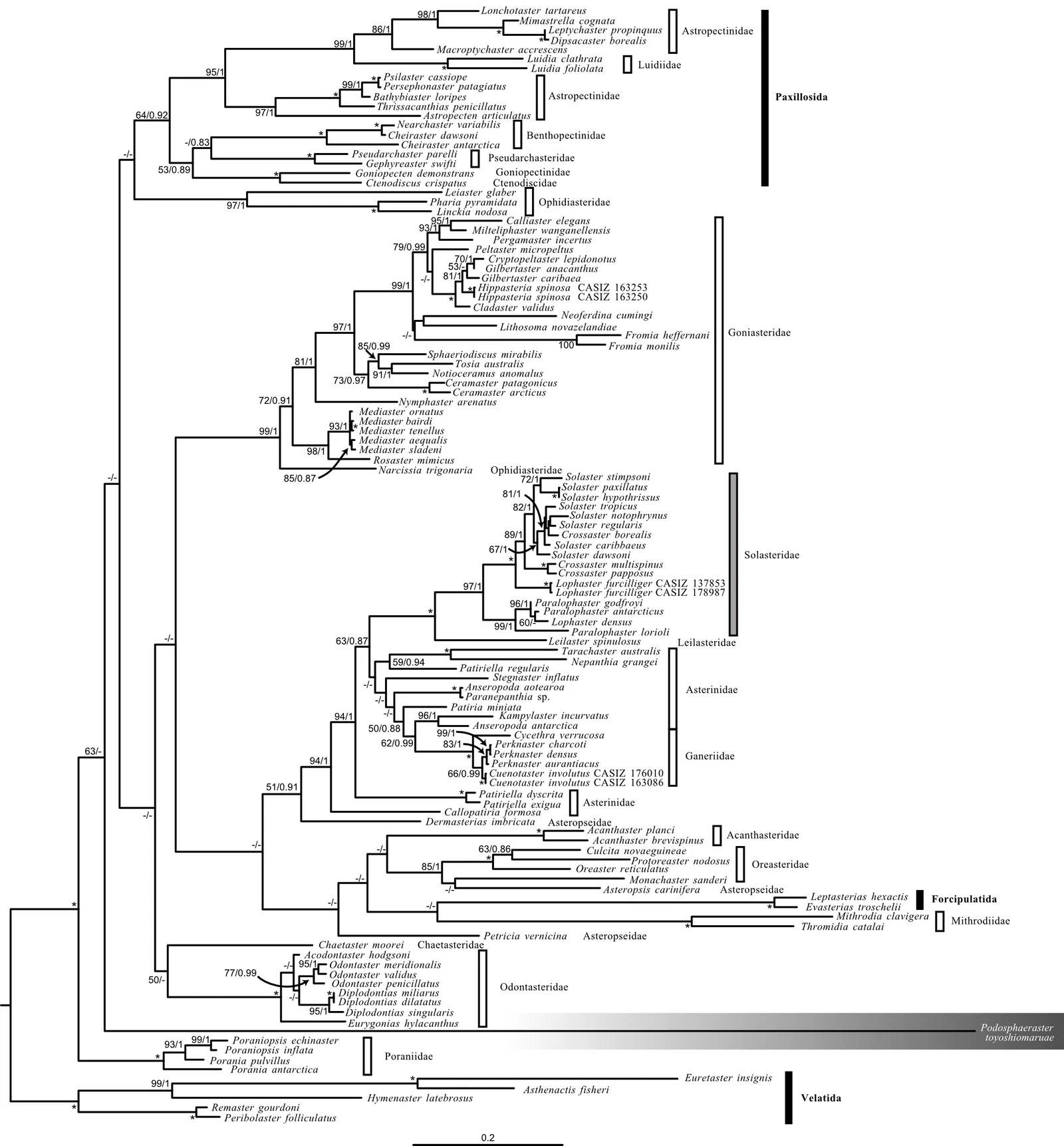
Maximum Likelihood tree inferred from the concatenated dataset of *rrnS*, *rrnL* and *histone H3* gene sequences. Numbers on nodes indicate bootstrap support (BS) values and posterior probabilities (PP). Asterisk (*) means 100% BS and 1.0 PP and hyphen (-) means below 50% BS or 0.80 PP. The unit of evolutionary distance is the number of base substitutions per site.

In our phylogenetic tree (Fig. 2, Suppl. material 1), the order Valvatida was resolved as polyphyletic. This is consistent with a finding by Mah and Foltz (2011) suggesting that Valvatida may encompass the order Paxillosida with extremely low support. Support for nodes branching higher taxa, including families within Valvatida, was generally low. *Podosphaeraster toyoshiomaruae* was positioned as the most basal lineage within Valvatida in our phylogeny; however, this placement received low statistical support in the Maximum Likelihood tree (bootstrap value = 63%) and no support in the Bayesian Inference tree (posterior probability < 0.80). The family Poraniidae was placed adjacent to *Podosphaeraster toyoshiomaruae*.

### Skeletal structure of Podosphaeraster toyoshiomaruae

**Figure 3.**
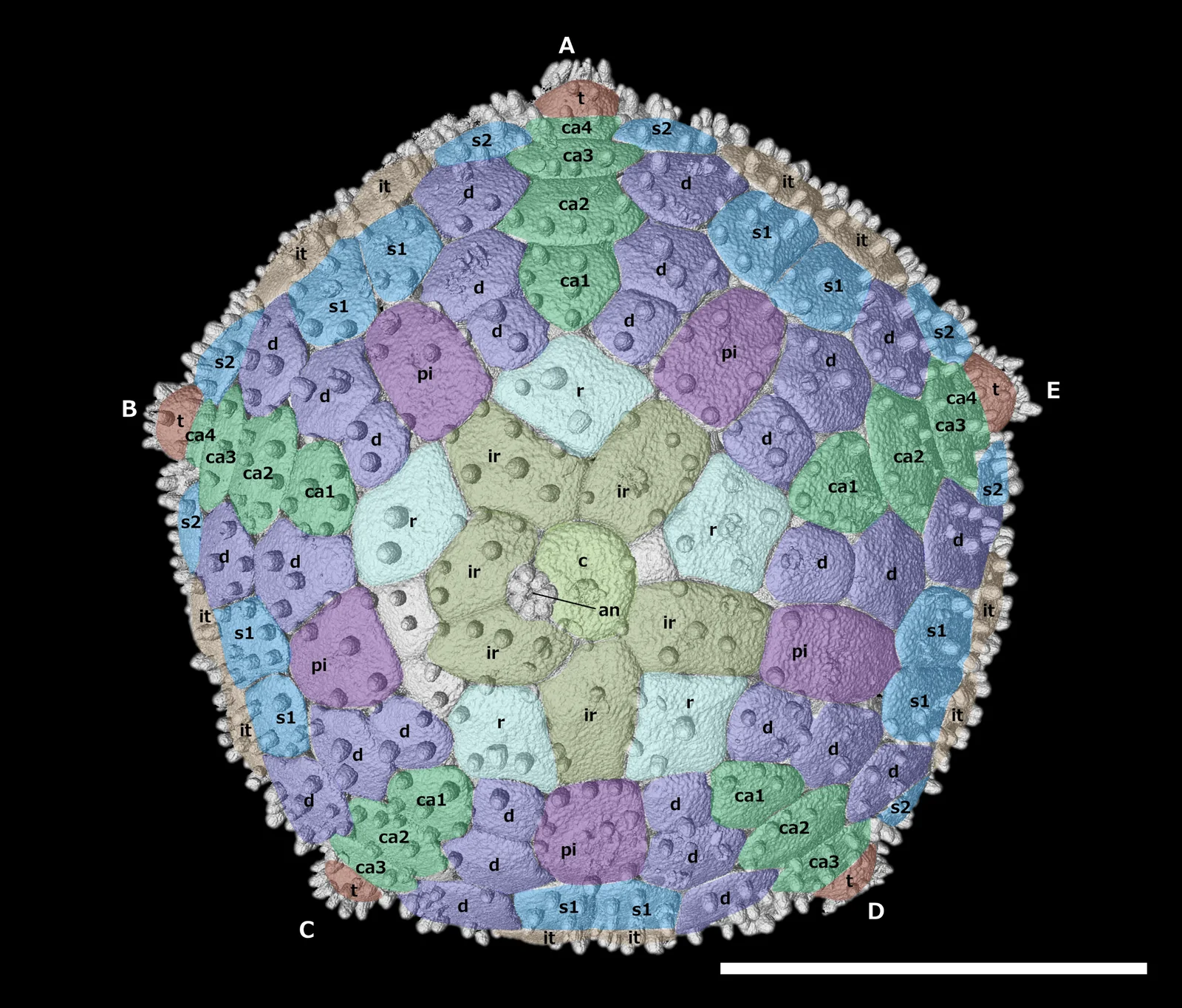
*Podosphaeraster toyoshiomaruae*. Micro-CT images of skeleton, NSMT E-13935A, External view of abactinal half. Scale bar: 5 mm. A–E, radii after Carpenter’s (1884) system. Abbreviations: a, actinal plate; ad, adambulacral series; am, ambulacral series; an, anus; c, central plate; ca; carinal plate; d, dorsolateral plate; i, I-plate; if, inferomarginal plate; ir, interradial plate; it, intermarginal plate; m, madreporite; od, odontophore; or, pair of oral plates; pi, primary interradial plate; r, radial plate; s, superomarginal plate; t, terminal plate; v, V-plate. Arabic numerals on the carinal, superomarginal and inferomarginal plates indicate plate numbers from proximal to distal.

**Figure 4.**
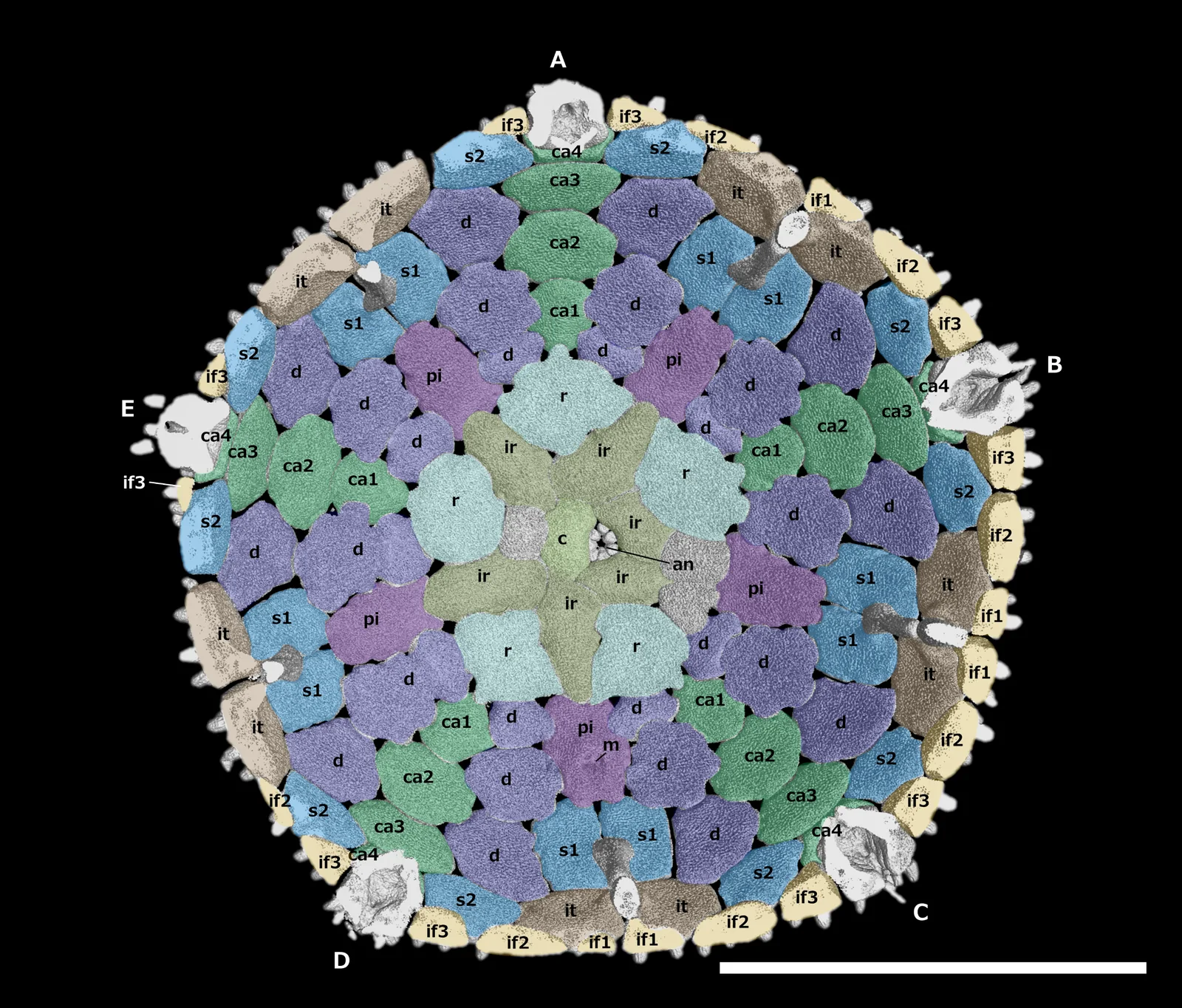
*Podosphaeraster toyoshiomaruae*. Micro-CT images of skeleton, NSMT E-13935A, Internal view of abactinal half. Scale bar: 5 mm. See Fig. 3 legend for arm labels and abbreviations of morphologies.

Body shape is sub-depressed with a horizontal diameter (HD) of 9.6 mm and a vertical diameter (VD) of 6.3 mm. The HD/VD ratio is 1.52. The apical system was polygonal, located at the abactinal centre and composed of one central, six interradial, five radial and three additional plates (Figs 3, 4). The central plate was oval and surrounded by six elongated interradial plates. These interradial plates were single in each interradius, except for two smaller plates in the interradius BC. Radial plates were wedge-shaped and alternately positioned between interradial plates. Outside this apical system, a longitudinally elongated primary interradial plate was present on each interradius. A series of four carinal plates was positioned between each pair of the radial and terminal plates, flanked by a longitudinal series of two or three dorsolateral plates. The first carinal plate was triangular, but the second to fourth plates were transversely elongated trapezoids. A madreporite was located at the centre of one of the primary interradial plates. An anus was surrounded by the central and two interradial plates in the interradius BC with coverage of seven granules.

**Figure 5.**
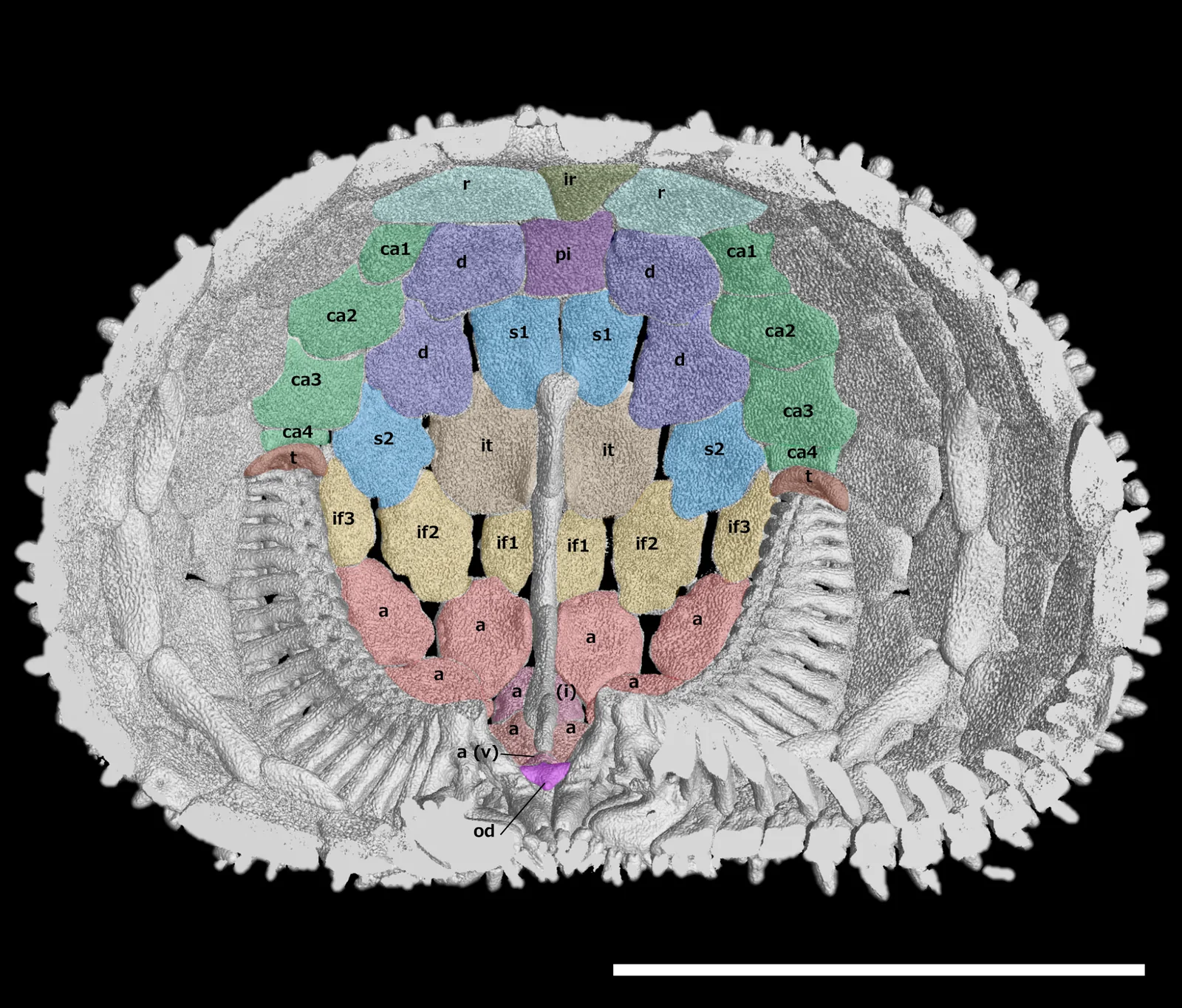
*Podosphaeraster toyoshiomaruae*. Micro-CT images of skeleton, NSMT E-13935A, Internal view of lateral half. Scale bar: 5 mm. See Fig. 3 legend for arm labels and abbreviations of morphologies.

Supero- and inferomarginal plates were four and six in each interradius, respectively and arranged in an arched series surrounding a pair of larger intermarginal plates (Fig. 5).

**Figure 6.**
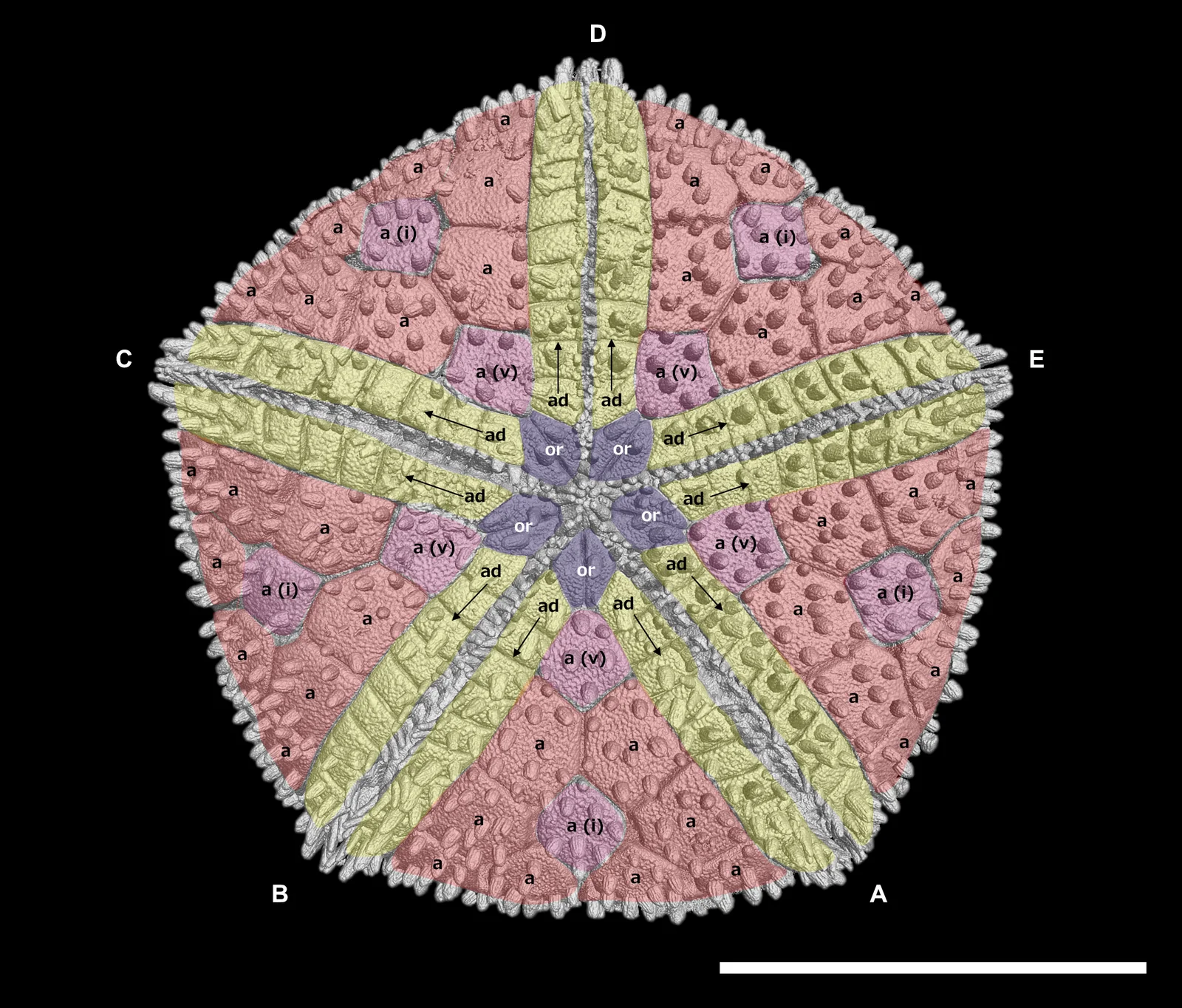
*Podosphaeraster toyoshiomaruae*. Micro-CT images of skeleton, NSMT E-13935A, External view of actinal half. Scale bar: 5 mm. See Fig. 3 legend for arm labels and abbreviations of morphologies.

**Figure 7.**
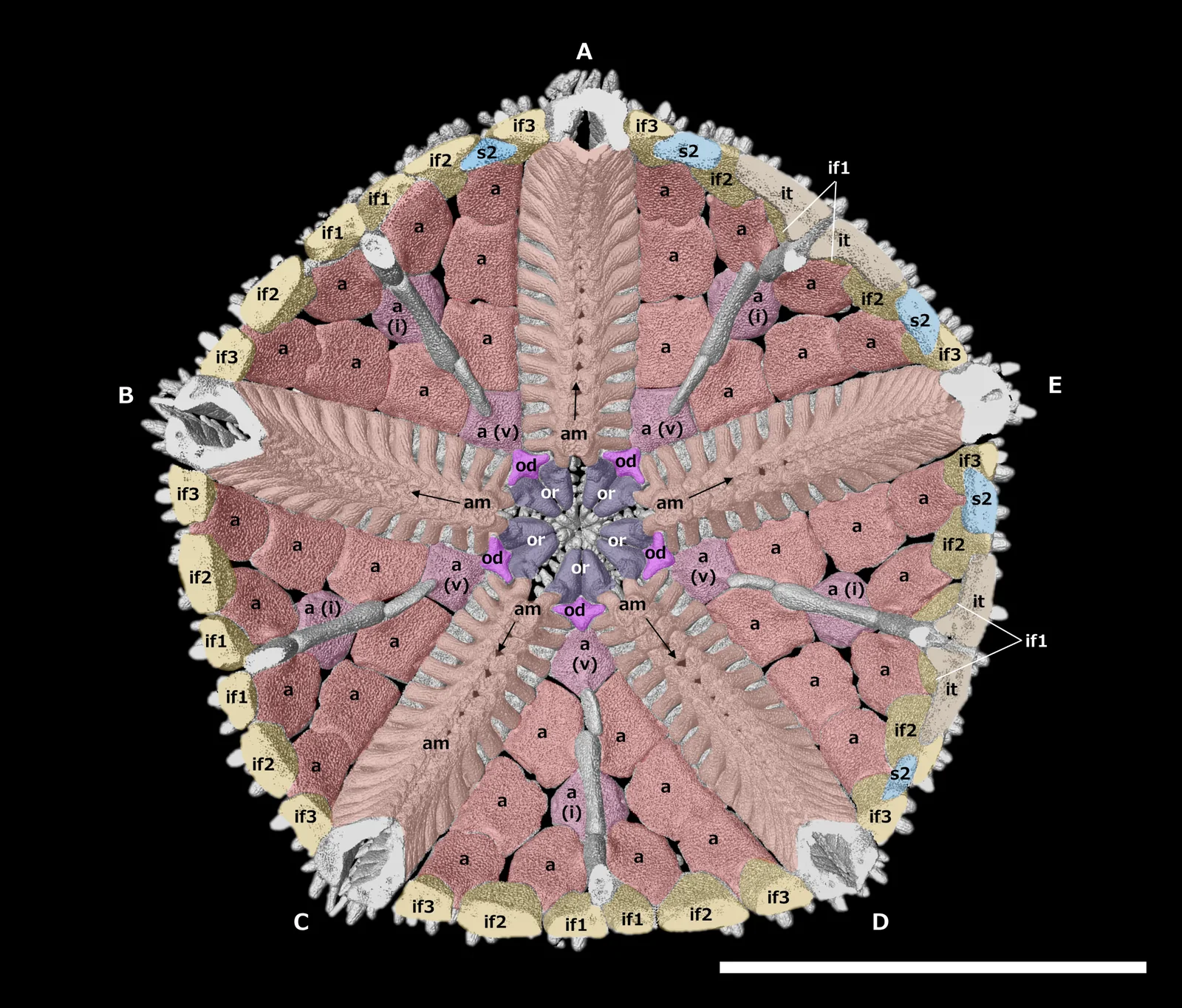
*Podosphaeraster toyoshiomaruae*. Micro-CT images of skeleton, NSMT E-13935A, Internal view of actinal half. Scale bar: 5 mm. See Fig. 3 legend for arm labels and abbreviations of morphologies.

Actinal plates were polygonal, 10 in each interradius (Figs 5, 6, 7). Two unpaired plates, I- and V-plates, were present in each interradius. The I-plate was surrounded by six other actinal plates and the V-plate was abutted by two actinal, six adambulacral and two oral plates. Each ambulacral groove was lined by two longitudinal series of 15 rectangular adambulacral plates and 17 ambulacral plates. The first ambulacral plates were Y-shaped, but the second to 17^th^ ambulacral plates were axe-shaped. Odontophores were quadrilobate and positioned between the neighbouring first ambulacral plates on the abactinal surface of the pair of triangular oral plates. In each interradius, four rod-shaped ossicles of the mesentery vertically lined the internal surface of the pairs of first superomarginal, intermarginal, first inferomarginal, actinal plates and a V-plate (Fig. 5). One of these four ossicles, which linked two pairs of actinal plates, was notched at the actinal surface and spanned the I-plate.

## Discussion

The highly specialised, spherical body of *Podosphaeraster toyoshiomaruae*, which is unique amongst asteroids, is mirrored by its significant genetic divergence. Although mitochondrial gene arrangements in echinoderms are generally highly conserved (Smith et al. 1993), recent mitogenome sequencing has increasingly revealed lineage-specific rearrangements, particularly in ophiuroids (Na et al. 2024). The mitogenome of *P. toyoshiomaruae* reported herein represents the first evidence of a major protein-coding gene translocation within Asteroidea (Fig. 1). This structural rearrangement is accompanied by high sequence divergence, including a unique initiation codon (ATT) for the *COB* gene, which typically utilises ATG in echinoderms (Quek et al. 2020).

Due to high mutation rates in the *rrnS*, *rrnL* and histone *H3* genes, *P. toyoshiomaruae* exhibited a conspicuously long branch in our phylogenetic analysis, potentially reducing nodal support across the tree via long-branch attraction (LBA). Overall, the three markers provided poor resolution for the deep phylogeny of asteroid higher taxa, likely because their evolutionary rates are too rapid to accurately resolve divergences that occurred approximately 250 million years ago (Gale 2011). Although substitution saturation tests indicated that the dataset is generally unsaturated at the family level (*Iss* < *Iss. c*), the difference between *Iss* and *Iss. c* was not significant under an asymmetric tree assumption with 16 OTUs l (Table 5), suggesting that saturation may confound relationships amongst specific taxa.

This phylogenetic instability is further complicated by the ongoing controversy surrounding outgroup selection and root placement in asteroid phylogenetics (Mah and Blake 2012, Feuda and Smith 2015). Following Feuda and Smith (2015), who suggested Velatida as an appropriate outgroup for their data and given that Velatida resolves basal to Valvatida in previous molecular studies (Janies et al. 2011, Mah and Foltz 2011), we provisionally utilised a Velatida clade to root our tree. Our resulting topology revealed several discrepancies with previous studies — such as Forcipulatacea forming a sister group with Mithrodiidae — but consistently supported the polyphyly of Valvatida. Confounded by LBA, the exact position of *P. toyoshiomaruae* remains unstable, preventing definitive resolution beyond its tentative placement as a basal lineage within Valvatida.

To resolve these deep relationships, future studies must employ a larger set of genetic markers with more conservative evolutionary rates. Additionally, discovering extant taxa closely related to the genus *Podosphaeraster* will be crucial to breaking this long branch and improving phylogenetic accuracy.

The micro-CT reconstructions revealed the arrangement and morphology of the skeletal plates (Figs 3, 4, 5, 6, 7). Although the examined specimen exhibited a higher number of interradial, dorsolateral and adambulacral plates than the type specimens, the characteristic arrangement of the abactinal, marginal and actinal plate series described by Fujita and Rowe (2002) was clearly observed.

Regarding the increased number of interradial plates, Fujita and Rowe (2002) interpreted a single plate on interradius BC as consisting of two parts divided by a bisecting longitudinal furrow. In contrast, our micro-CT observations revealed that these are two independent plates. We suggest that this discrepancy is an artefact of the observation method rather than a biological difference between the samples.

The relatively high HD/VD ratio (approximately 1.5) observed in our micro-CT specimen likely resulted from the physiological state during fixation, such as the effects of anaesthesia. Ecological observations (Yonetani et al. 2017) indicate that the aspect ratio of this species can change during locomotion, particularly when the tube feet are extended. This value does not represent a significant departure from the 1.1–1.2 ratio observed in the type series.

However, due to the limited number of specimens and the difficulty of DNA extraction from formalin-fixed samples in previous studies, it remains unclear whether these differences reflect morphological or genetic variation. Further studies with additional samples are required to resolve this issue.

The presence of calcified reinforcement in the mesentery of *P. toyoshiomaruae* had been previously implied (referred to as an "interradial ridge" by Fujita and Rowe (2002)), but the detailed structure of its skeleton is presented here for the first time (Fig. 5). The micro-CT images revealed that the mesentery skeleton spans the I-plate and connects to the V-plate and five pairs of plates, including three types of marginal plates. This observation suggests that the marginal plates (*sensu* Fujita and Rowe (2002)) of this species may not be homologous to those of other asteroids, as the mesentery skeleton in other asteroids is typically contiguous with abactinal/actinal plates and odontophores, but not with marginal plates (Gale 2011). Notably, while the connectivity of the mesentery skeleton varies, there is a lack of significant morphological differentiation between the marginal plate series and the adjacent dorsolateral and actinal plates, as shown by micro-CT imaging. Gale (2021) also questioned the homology of marginal plates in *Podosphaeraster*, suggesting that Sphaerasteridae evolved from the Mesozoic family Stauranderasteridae through the development of a spherical body and the subsequent loss of differentiated marginal plates. Although varying degrees of marginal plate reduction are shared with the order Velatida, we consider this to be a case of convergent evolution. Our phylogenetic analysis indicates a close relationship between *P. toyoshiomaruae* and Poraniidae (and other Valvatida), all of which possess marginal plates differentiated to varying extents, the homology of which remains uncertain.

Strictly speaking, the evolutionary origins of these marginal plates remain unknown without comparative evo-devo studies. Therefore, to maintain consistency regarding relative positions, we have adopted the terminology used by Fujita and Rowe (2002) for all anatomical features, including the apical system. Notably, unlike the examined *P. toyoshiomaruae*, Gale (2021) reported an internal swelling of the I-plate, potentially associated with vertical interradial support, in one of its putative relatives, the extinct Eosphaeraster. More observations of mesenteric skeletons using micro-CT of various *Podosphaeraster* species will help to assess the homology and diversity of skeletal ossicles amongst Podosphaerasteridae taxa.

## Conclusions

*Podosphaeraster toyoshiomaruae* has unique morphological characters amongst asteroids, which are mirrored by significant genetic divergence. Despite conservative evolution of the asteroidean mitochondrial genome, a *COX2* gene translocation was observed. *P. toyoshiomaruae* appears to evolve relatively faster than other asteroidean species, which resulted in an ambiguous phylogenetic position likely caused by LBA. Therefore, to elucidate a more robust phylogeny, it will be necessary to utilise a larger set of genetic markers with more appropriate evolutionary rates. Furthermore, the discovery of as-yet-unknown species more closely related to the genus *Podosphaeraster* would be crucial for resolving the long branch and improving phylogenetic accuracy.

Our morphological observation using micro-CT suggested that the marginal plates of this species were not homologous with those of other asteroids. Although the phylogenetic analysis was not robust, it indicated a close relationship between *P. toyoshiomaruae* and Poraniidae and other Valvatida, which share differentiated marginal plates to varying extents.

More observations of mesenteric skeletons using micro-CT of various *Podosphaeraster* species will help to assess the homology and diversity of skeletal ossicles amongst Podosphaerasteridae taxa.

## Supplementary Material

907BA487-7434-506F-ADB0-E38FC8F3AE85 10.3897/BDJ.14.e194006.suppl1 Supplementary material 1 Bayesian tree inferred from the concatenated dataset of *rrnS*, *rrnL* and *histone H3* gene sequences Data type image Brief description Bayesian tree inferred from the concatenated dataset of *rrnS*, *rrnL* and *histone H3* gene sequences. Nodal numbers indicate posterior probabilities (PP). The unit of evolutionary distance is the number of base substitutions per site. File: oo_1570870.png https://binary.pensoft.net/file/1570870 SF Hiruta, I Kobayashi, T Fujita

## Figures and Tables

**Table 1.** Species list for phylogenetic analysis with accession numbers. Abbreviations: *1, Mah and Foltz (2011); *2, Foltz and Mah (2010); *3, Janies et al. (2011); *4, Yasuda et al. (2006); *5, Waters et al. (2004).

Order	Family	Species	Voucher	Accession No.			Source
				*rrnS*	*rrnL*	*histone H3*	
Valvatida	Podosphaerasteridae	*Podosphaeraster toyoshiomaruae*	NSMT E-14365A	LC913548		LC913551	This study
	Acanthasteridae	*Acanthaster brevispinus*		AB231476	AB231476		*4
		*Acanthaster planci*		AB231475	AB231475		*4
	Asterinidae	*Anseropoda antarctica*	CASIZ 175766	EU723018	EU722940	EU707722	*1
		*Anseropoda aotearoa*	NIWA 15416	EU723078	EU722997	EU707755	*1
		*Callopatiria formosa*	CASIZ 102191	GQ288476	GQ288521	GQ288581	*2
		*Kampylaster incurvatus*	CASIZ 167617	GQ288477	GQ288522	GQ288571	*2
		*Nepanthia grangei*	NIWA 15417	EU723079	EU722998	EU707753	*1
		*Paranepanthia* sp.	MNHNP EcAs 12508	GQ288471	GQ288516	GQ288566	*2
		*Patiria miniata*	AMCC 113402		DQ297074	DQ676897	*3
		*Patiriella dyscrita*	CASIZ 118456	GQ288473	GQ288518	GQ288574	*2
		*Patiriella exigua*	CASIZ 117900	EU723040	EU722963	EU707724	*1
		*Patiriella regularis*	AMCC 113390	DQ273733		DQ676925	*3
		*Stegnaster inflatus*		EU723076	EU722995	EU707756	*1
		*Cycethra verrucosa*		EU723017	EU722939	EU707654	*1
		*Tarachaster australis*	NIWA 15419	EU723061	EU722983	EU707754	*1
	Asteropseidae	*Dermasterias imbricata*		AY370691	AY370718		*5
		*Asteropsis carinifera*	CASIZ 117863	EU072964	EU072935	EU707689	*1
		*Petricia vernicina*	NIWA 43672	EU723077	EU722996	EU707757	*1
	Chaetasteridae	*Chaetaster moorei*	CASIZ 118047	EU072967	EU072938	EU707672	*1
	Ganeriidae	*Perknaster aurantiacus*	CASIZ 174670	GQ288493	GQ288554	GQ288585	*2
		*Perknaster charcoti*	CASIZ 163085	EU072985	EU072956	EU707686	*1
		*Perknaster densus*	CASIZ 174651	GQ288494	GQ288555	GQ288586	*2
		*Cuenotaster involutus*	CASIZ 163086	EU072970	EU072941	EU707671	*1
		*Cuenotaster involutus*	CASIZ 176010	GQ288492	GQ288553	GQ288582	*2
	Goniasteridae	*Calliaster elegans*	MNHNP EcAh 4845	EU723050	EU722973	EU707726	*1
		*Ceramaster arcticus*	CASIZ 143443	EU723021	EU722943	EU707731	*1
		*Ceramaster patagonicus*	CASIZ 174590	EU723035	EU722958	EU707732	*1
		*Cladaster validus*	CASIZ 158252	EF624432	EF624400	EU707669	*1
		*Cryptopeltaster lepidonotus*	CASIZ 111828	GQ288486	GQ288531	GQ288580	*2
		*Fromia heffernani*	CASIZ 113789	EU723020	EU722942	EU707730	*1
		*Fromia monilis*	CASIZ 107520	EU072975	EU072945	EU707692	*1
		*Neoferdina cumingi*	CASIZ 113512	EU723029	EU722951	EU707729	*1
		*Gilbertaster anacanthus*	CASIZ 159080	GQ288478	GQ288523	GQ288568	*2
		*Hippasteria caribaea*	USNM 1126238	GQ288485	GQ288530	GQ288604	*2
		*Hippasteria spinosa*	CASIZ 163253	EU723022	EU722944	EU707728	*1
		*Hippasteria spinosa*	CASIZ 163250	GQ288482	GQ288525	GQ288576	*2
		*Lithosoma novaezelandiae*	NIWA 15423	EU723068	EU722989	EU707749	*1
		*Mediaster aequalis*	CASIZ 120090	EU723013	EU072953	EU707662	*1
		*Mediaster bairdi*	USNM 1127106	GQ288489	GQ288535	GQ288601	*2
		*Mediaster ornatus*	CASIZ 157499	EU723047	EU722970	GQ288562	*2
		*Mediaster sladeni*	NIWA 27627	EU723069	EU722990	EU707751	*1
		*Mediaster tenellus*	CASIZ 178975	GQ288488	GQ288534	GQ288594	*2
		*Milteliphaster wanganellensis*	CASIZ 108646	EU723025	EU722947	EU707725	*1
		*Notioceramus anomalus*	CASIZ 176007	GQ288481	GQ288527	GQ288577	*2
		*Nymphaster arenatus*	CASIZ 112827	GQ288484	GQ288526	GQ288579	*2
		*Peltaster micropeltus*	CASIZ 171710	EU723030	EU722953	EU707727	*1
		*Pergamaster incertus*	CASIZ 163059	GQ288483	GQ288529	GQ288578	*2
		*Pseudarchaster parelii*	CASIZ 120380	EU723042	EU722965	EU707737	*1
		*Rosaster mimicus*	NIWA 15432	EU723059	EU722980	EU707750	*1
		*Sphaeriodiscus mirabilis*	CASIZ 174584	GQ288487	GQ288533	GQ288606	*2
		*Tosia australis*	USNM 1135012	EU072990	EU072960	EU707693	*1
	Leilasteridae	*Leilaster spinulosus*	MNHNP EcAs 12610	GQ288469	GQ288514	GQ288565	*2
	Mithrodiidae	*Mithrodia clavigera*	CASIZ 115525	EU072983	EU072954	EU707699	*1
		*Thromidia catalai*	CASIZ 107266	EU072989	EU072959	EU707698	*1
	Odontasteridae	*Acodontaster hodgsoni*	CASIZ 174598	EU072963	EU072933	EU707685	*1
		*Diplodontias dilatatus*	AMCC 113383	DQ273732	DQ297075	DQ676898	*3
		*Diplodontias miliaris*	AMCC 113392	DQ377827	DQ297078	DQ676900	*3
		*Diplodontias singularis*		EU723016	EU722938	EU707653	*1
		*Eurygonias hylacanthus*	AMCC 114324	DQ273739	DQ297089	DQ676908	*3
		*Odontaster meridionalis*	AMCC 113406	DQ273730	DQ297100	DQ676917	*3
		*Odontaster penicillatus*		EU723001	EU722926	EU707656	*1
		*Odontaster validus*	CASIZ 169926	EF624444	EF624414	EU707663	*1
	Ophidiasteridae	*Leiaster glaber*	CASIZ 104486	EU723023	EU722945	EU707748	*1
		*Linckia nodosa*	CASIZ 112840	EU723024	EU722946	EU707734	*1
		*Narcissia trigonaria*	USNM 1127111	GQ288490	GQ288537	GQ288603	*2
		*Pharia pyramidata*	CASIZ 163788	EU723033	EU722956	EU707759	*1
	Oreasteridae	*Culcita novaeguineae*	CASIZ 113508	EU072971	EU072942	EU707688	*1
		*Monachaster sanderi*	CASIZ 173553	EU723026	EU722948	EU707733	*1
		*Oreaster reticulatus*	USNM 1135008	GQ288470	GQ288515	GQ288570	*2
		*Protoreaster nodosus*	USNM1135007	GQ288472	GQ288517	GQ288569	*2
	Poraniidae	*Porania antarctica*	CASIZ 167616	EU072986	EF624418	EU707664	*1
		*Porania pulvillus*	USNM 1127105	EU722999	EU722924	EU707658	*1
		*Poraniopsis echinaster*		EU723000	EU722923	EU707652	*1
		*Poraniopsis inflata*	CASIZ 120150	EU723041	EU722964	EU707741	*1
	Solasteridae	*Crossaster borealis*	CASIZ 179002	GQ288502	GQ288543	GQ288589	*2
		*Crossaster multispinus*	NIWA 27595	GQ288468	GQ288513	GQ288567	*2
		*Crossaster papposus*	AMCC 113349	DQ273725	DQ297084	DQ676904	*3
		*Lophaster densus*	CASIZ 163121	EU072980	EU072950	EU707666	*1
		*Lophaster furcilliger*	CASIZ 137853	GQ288475	GQ288520	GQ288572	*2
		*Lophaster furcilliger*	CASIZ 178987	GQ288504	GQ288550	GQ288592	*2
		*Paralophaster antarcticus*	CASIZ 174645	GQ288506	GQ288552	GQ288588	*2
		*Paralophaster godfroyi*	CASIZ 176011	GQ288505	GQ288551	GQ288587	*2
		*Paralophaster lorioli*	CASIZ 167622	EU723058	EU722979	GQ288563	*2
		*Solaster caribbaeus*	USNM 1127108	GQ288501	GQ288549	GQ288602	*2
		*Solaster dawsoni*	CASIZ 119165	GQ288474	GQ288519	GQ288573	*2
		*Solaster hypothrissus*	CASIZ 163958	GQ288496	GQ288546	GQ288584	*2
		*Solaster notophrynus*	CASIZ 163147	GQ288499	GQ288545	GQ288583	*2
		*Solaster paxillatus*	CASIZ 164100	GQ288497	GQ288547	GQ288590	*2
		*Solaster regularis*	CASIZ 174602	GQ288503	GQ288544	GQ288591	*2
		*Solaster stimpsoni*	AMCC 113400	DQ273726	DQ297113	DQ676930	*3
		*Solaster tropicus*	CASIZ 104211	GQ288500	GQ288542	GQ288593	*2
Paxillosida	Astropectinidae	*Astropecten articulatus*	AMCC 113394	DQ273741	DQ297079	DQ676901	*3
		*Bathybiaster loripes*	CASIZ 162501	EU072966	EU072937	EU707657	*1
		*Dipsacaster borealis*	CASIZ 143420	EU723037	EU722960	EU707738	*1
		*Leptychaster propinquus*	CASIZ 158249	EU723039	EU722962	EU707739	*1
		*Lonchotaster tartareus*	CASIZ 163084	EU072979	EU072949	EU707660	*1
		*Macroptychaster accrescens*	AMCC 113410	DQ273742	DQ297098	DQ676915	*3
		*Mimastrella cognata*		EU723005	EU722928	EU707655	*1
		*Persephonaster patagiatus*	CASIZ 116778	EU723006	EU722937	EU707650	*1
		*Psilaster cassiope*	CASIZ 178991	GQ288511	GQ288540	GQ288600	*2
		*Thrissacanthias penicillatus*	CASIZ 115075	EU723045	EU722968	EU707740	*1
	Benthopectinidae	*Cheiraster antarcticus*	CASIZ 169932	EU072981	EU072951	EU707641	*1
		*Cheiraster dawsoni*	CASIZ 143965	EU723028	EU722950	EU707736	*1
		*Nearchaster variabilis*	CASIZ 178988	GQ288495	GQ288538	GQ288595	*2
	Ctenodiscidae	*Ctenodiscus crispatus*	CASIZ 173281	EU072969	EU072940	EU707696	*1
	Goniopectinidae	*Goniopecten demonstrans*		GQ288507	GQ288539	GQ288605	*2
	Luidiidae	*Luidia clathrata*	AMCC 113393	DQ273743	DQ297096	DQ676913	*3
		*Luidia foliolata*	CASIZ 105625	EU072982	EU072952	EU707697	*1
	Pseudarchasteridae	*Gephyreaster swifti*	CASIZ 158251	EU072976	EU072946	EU707690	*1
Velatida	Korethrasteridae	*Remaster gourdoni*	CASIZ 167615	EU072987	EU072957	EU707667	*1
		*Peribolaster folliculatus*	CASIZ 163123	EU072984	EU072955	EU707668	*1
	Myxasteridae	*Asthenactis fisheri*	CASIZ 157505	EU723034	EU722957	EU707746	*1
	Pterasteridae	*Euretaster insignis*	CASIZ 104210	EU072974	EU072944	EU707691	*1
		*Hymenaster latebrosus*	CASIZ 163076	EU072978	EU072948	EU707670	*1
Forcipulatida	Asteriidae	*Evasterias troschelii*	AMCC 113398		DQ297090	DQ676909	*3
		*Leptasterias hexactis*	AMCC 113404	DQ777079	DQ297094	DQ676912	*3

**Table 2.** Best partition schemes and corresponding best models of substitution for the concatenated dataset as determined with Partition Finder.

Partition	Model for ML	Model for BA	Gene	Region
1	GTR+I+G4	GTR+I+G4	*rrnS*	1-1290
2	GTR+I+G4	GTR+I+G4	*rrnL*	1291-2867
3	TVMef+I+G4	GTR+I+G4	*histone H3*	2868-3203

**Table 3.** Mitochondrial genome organisation of the *Podosphaeraster toyoshiomaruae*.

Gene	Strand	Begins	Ends	Length	3' spacer	Start codon	Stop codon	Anticodon
*COX1*	+	1	1566	1566	10	ATG	TAG	
*trnR*	+	1557	1621	65	-10			TCG
*NAD4L*	+	1622	1918	297	0	ATT	TAA	
*trnK*	+	1929	1999	71	10			CTT
*ATP8*	+	2002	2163	168	2	ATG	TAA	
*ATP6*	+	2172	2837	666	8	ATG	TAA	
*COX3*	+	2852	3634	783	14	ATG	TAA	
*trnS2*	-	3635	3706	72	0			TGA
*NAD3*	+	3714	4064	351	7	ATT	TAA	
*NAD4*	+	4072	5448	1377	7	ATG	TAG	
*COX2*	+	5473	6162	690	24	ATG	TAA	
*trnH*	+	6203	6274	72	40			GTG
*trnS1*	+	6319	6386	68	44			GCT
*NAD5*	+	6387	8204	1818	0	ATG	TAA	
*NAD6*	-	8217	8711	495	12	ATG	TAA	
*COB*	+	8748	9896	1149	36	ATT	TAG	
*trnF*	+	9905	9973	69	8			GAA
*rrnS*	+	9975	10858	884	1			
*trnE*	+	10856	10925	70	-3			TTC
*trnT*	+	10935	11002	68	9			TGT
control region		11003	11443	441	0			
*rrnL*	-	11444	13024	1581	0			
*NAD2*	-	13143	14147	1005	118	ATG	TAA	
*trnI*	-	14205	14275	71	57			GAT
*NAD1*	-	14275	15246	972	-1	ATG	TAG	
*trnL2*	-	15253	15324	72	6			TAA
*trnG*	-	15338	15408	71	13			TCC
*trnY*	-	15416	15484	69	7			GTA
*trnD*	+	15489	15559	71	4			GTC
*trnM*	-	15563	15634	72	3			CAT
*trnV*	+	15644	15711	68	9			TAC
*trnC*	-	15718	15786	69	6			GCA
*trnW*	-	15786	15852	67	-1			TCA
*trnA*	+	15867	15935	69	14			TGC
*trnL1*	-	15939	16008	70	3			TAG
*trnN*	-	16011	16082	72	2			GTT
*trnQ*	+	16084	16158	75	1			TTG
*trnP*	-	16169	16237	69	10			TGG

**Table 4.** Codon usage in the 13 mitochondrial PCGs of *Podosphaeraster toyoshiomaruae*. RSCU: relative synonymous codon usage.

Amino acid	Codon	Frequency	RSCU	Amino acid	Codon	Frequency	RSCU
Phe	UUU	203	1.35	Ser2	UCU	116	2.64
	UUC	97	0.65		UCC	82	1.74
Leu2	UUA	187	1.90		UCA	72	1.52
	UUG	51	0.52		UCG	10	0.21
Leu1	CUU	135	1.37	Pro	CCU	53	1.2
	CUC	86	0.87		CCC	32	0.72
	CUA	123	1.25		CCA	87	1.97
	CUG	10	0.10		CCG	5	0.11
Ile	AUU	206	1.36	Thr	ACU	74	1.17
	AUC	87	0.58		ACC	74	1.17
	AUA	160	1.06		ACA	99	1.57
Met	AUG	85	1.00		ACG	5	0.08
Val	GUU	82	1.59	Ala	GCU	74	1.48
	GUC	27	0.52		GCC	56	1.12
	GUA	70	1.36		GCA	65	1.3
	GUG	27	0.52		GCG	5	0.1
Tyr	UAU	69	1.12	Cyc	UGU	18	1.06
	UAC	54	0.88		UGC	16	0.94
Stop	UAA	9	1.38	Trp	UGA	77	1.59
	UAG	4	0.62		UGG	20	0.41
His	CAU	38	0.93	Arg	CGU	13	0.85
	CAC	44	1.07		CGC	5	0.33
Gln	CAA	79	1.82		CGA	40	2.62
	CAG	8	0.18		CGG	3	0.2
Asn	AAU	75	0.97	Ser1	AGU	19	0.4
	AAC	45	0.58		AGC	11	0.23
	AAA	111	1.44		AGA	56	1.19
Lys	AAG	54	1.00		AGG	12	0.25
Asp	GAU	30	0.97	Gly	GGU	68	1.25
	GAC	32	1.03		GGC	32	0.59
Glu	GAA	62	1.72		GGA	89	1.63
	GAG	10	0.28		GGG	29	0.53

**Table 5.** Results of the substitution saturation test for the dataset comprising *rrnS*, *rrnL* and *histone H3* genes. Two-tailed t-tests were used to compare the observed index of substitution saturation (*Iss*) against the critical indices. *Iss.c_Sym* and *Iss.c_Asym* represent the critical indices of substitution saturation assuming symmetrical and asymmetrical tree topologies, respectively. Degrees of freedom for all tests were 1,494.

Number of OTUs	*Iss*	*Iss.c_Sym*	T	P	*Iss.c_Asym*	T	P
4	0.503	0.833	17.222	0.0000	0.803	15.644	0.0000
8	0.554	0.809	10.237	0.0000	0.709	6.219	0.0000
16	0.610	0.792	5.854	0.0000	0.609	0.039	0.9692
32	0.672	0.774	2.823	0.0048	0.491	4.990	0.0000
